# Selective omission of sentinel lymph node biopsy in mastectomy for ductal carcinoma in situ: identifying eligible candidates

**DOI:** 10.1186/s13058-024-01816-7

**Published:** 2024-04-12

**Authors:** Soong June Bae, Yoonwon Kook, Ji Soo Jang, Seung Ho Baek, Sohyun Moon, Jung Hyun Kim, Seung Eun Lee, Min Ji Kim, Sung Gwe Ahn, Joon Jeong

**Affiliations:** 1 Department of Surgery, Gangnam Severance Hospital, Yonsei University College of Medicine, Seoul, Republic of Korea; 2 Institute for Breast Cancer Precision Medicine, Yonsei University College of Medicine, Seoul, Republic of Korea

## Abstract

**Background:**

Sentinel lymph node biopsy (SLNB) is recommended for patients with ductal carcinoma in situ (DCIS) undergoing mastectomy, given the concerns regarding upstaging and technical difficulties of post-mastectomy SLNB. However, this may lead to potential overtreatment, considering favorable prognosis and de-escalation trends in DCIS. Data regarding upstaging and axillary lymph node metastasis among these patients remain limited.

**Methods:**

We retrospectively reviewed patients with DCIS who underwent mastectomy with SLNB or axillary lymph node dissection at Gangnam Severance Hospital between January 2010 and December 2021. To explore the feasibility of omitting SLNB, we assessed the rates of DCIS upgraded to invasive carcinoma and axillary lymph node metastasis. Binary Cox regression analysis was performed to identify clinicopathologic factors associated with upstaging and axillary lymph node metastasis.

**Results:**

Among 385 patients, 164 (42.6%) experienced an invasive carcinoma upgrade: microinvasion, pT1, and pT2 were confirmed in 53 (13.8%), 97 (25.2%), and 14 (3.6%) patients, respectively. Seventeen (4.4%) patients had axillary lymph node metastasis. Multivariable analysis identified age ≤ 50 years (adjusted odds ratio [OR], 12.73; 95% confidence interval [CI], 1.18–137.51; *p* = 0.036) and suspicious axillary lymph nodes on radiologic evaluation (adjusted OR, 9.31; 95% CI, 2.06–41.99; *p* = 0.004) as independent factors associated with axillary lymph node metastasis. Among patients aged > 50 years and/or no suspicious axillary lymph nodes, only 1.7–2.3%) experienced axillary lymph node metastasis.

**Conclusions:**

Although underestimation of the invasive component was relatively high among patients with DCIS undergoing mastectomy, axillary lymph node metastasis was rare. Our findings suggest that omitting SLNB may be feasible for patients over 50 and/or without suspicious axillary lymph nodes on radiologic evaluation.

**Supplementary Information:**

The online version contains supplementary material available at 10.1186/s13058-024-01816-7.

## Introduction

Ductal carcinoma in situ (DCIS) is a noninvasive neoplastic lesion of the breast, comprising approximately 25% of all newly diagnosed breast cancers [1–3]. As DCIS is characterized by the proliferation of malignant epithelial cells confined within the basement membrane [3], patients with DCIS who receive appropriate treatment have an excellent prognosis. According to a previous study assessing over 100,000 patients with DCIS from the Surveillance, Epidemiology, and End Results database, the 20-year breast cancer mortality was only 3.3% [4]. Furthermore, the risk of ipsilateral invasive recurrence at 20 years was 5.9%, and that of contralateral invasive recurrence at 20 years was 6.2%. Despite these characteristics, mastectomy has been performed in at least 20% of patients with DCIS, especially those with extensive or multifocal/multicentric lesions [4].

Axillary lymph node metastasis has long been considered a critical prognostic factor to guide systemic therapy or radiotherapy in patients with invasive breast cancer [5]. Sentinel lymph node biopsy (SLNB) is currently the standard surgical procedure to determine axillary staging [6, 7]. To ameliorate surgical complications and improve the patient’s quality of life by reducing axillary intervention, several ongoing prospective randomized trials are exploring the possibility of omitting SLNB in early breast cancer [8, 9].

Regarding axillary surgery in DCIS, SLNB is unessential in most patients with pure DCIS undergoing breast-conserving surgery. Conversely, SLNB is strongly recommended for patients diagnosed with DCIS requiring mastectomy owing to the following concerns [3]: (i) patients with DCIS who undergo a mastectomy have a high probability of upgrading to invasive breast cancer, and (ii) mastectomy can permanently alter the lymphatic drainage pattern, hampering the performance of additional SLNB if invasive breast cancer is confirmed unexpectedly in patients who had undergone mastectomy alone. We hypothesized that a substantial portion of patients diagnosed with DCIS and requiring mastectomy could potentially omit SLNB. However, limited data exists regarding the incidence of upgrade to invasive breast cancer and axillary lymph node metastasis in patients diagnosed with DCIS who underwent mastectomy.

This study aimed to assess the prevalence of upgrade to invasive breast cancer and axillary lymph node metastasis in patients who were diagnosed with DCIS on biopsy and subsequently underwent mastectomy with axillary surgery to establish the need for SLNB. Furthermore, we explored the clinicopathologic features related to the upgrade to invasive breast cancer and axillary lymph node metastasis.

## Methods

### Study population

The study protocol was reviewed and approved by the Institutional Review Board of the Gangnam Severance Hospital, Yonsei University, Seoul, Korea (IRB no. 3-2023-0026), and adhered to the tenets of the Declaration of Helsinki. The requirement for written informed consent was waived owing to the retrospective study design. The study was registered as a retrospective study on ClinicalTrials.gov, trial number NCT05961280.

**Fig. 1:**
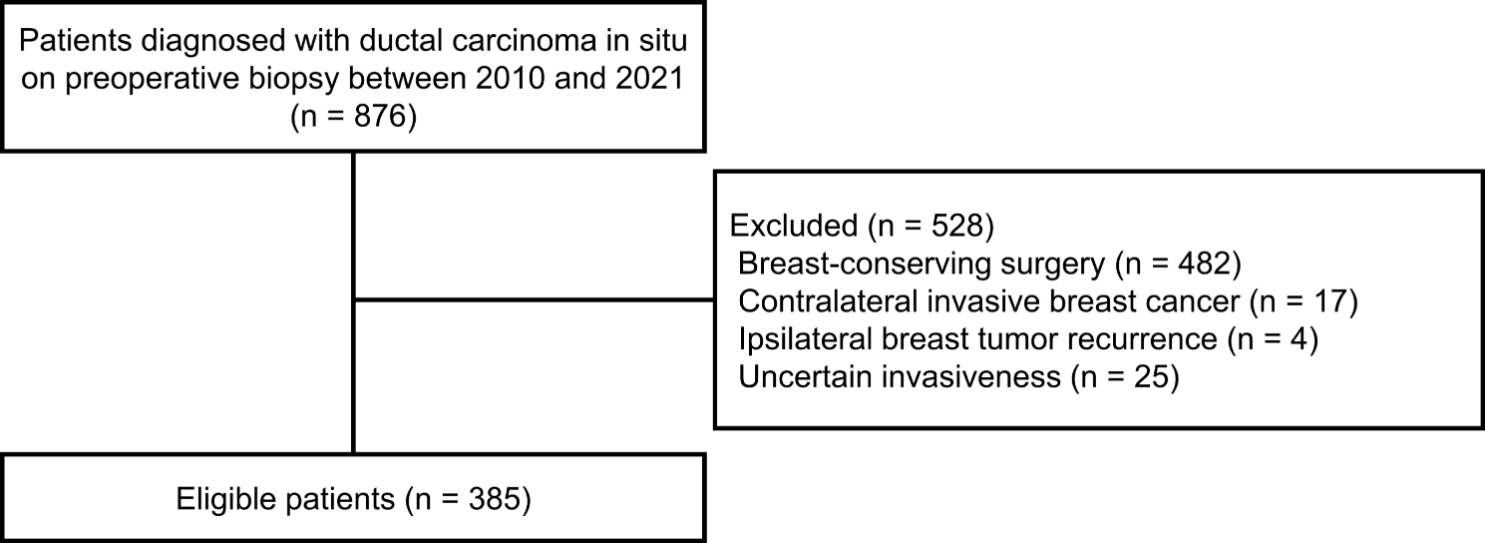
Flow diagram of the study population

Between January 2010 and December 2021, we retrospectively identified 876 women diagnosed with DCIS in preoperative biopsy samples obtained by core needle biopsy, vacuum-assisted breast biopsy, or excisional biopsy and subsequently underwent curative surgery. Of these, we excluded 528 women who (1) received breast-conserving surgery (*n* = 482), (2) had concurrent contralateral invasive breast cancer (*n* = 17), (3) were in case of ipsilateral breast tumor recurrence (*n* = 4), and (4) whose invasiveness was uncertain in the biopsy samples (*n* = 25). Finally, 385 patients were included retrospectively (Fig. 1). All patients underwent mastectomy with axillary surgery (SLNB or SLNB with subsequent axillary lymph node dissection [ALND]).

### Clinicopathologic features

We reviewed the electronic medical records to collect pre- and postoperative patient characteristics. Preoperative characteristics included age at diagnosis, presenting clinical symptoms (palpable mass or bloody nipple discharge), radiologic findings (clinical tumor size and the presence of suspicious axillary lymph node and microcalcification), and the pathologic findings from the biopsy samples (nuclear grade and comedo necrosis). Postoperative characteristics included pathologic findings from surgical specimens, such as pathologic DCIS size; nuclear grade (NG); comedo necrosis; status of estrogen receptor (ER), progesterone receptor (PR), human epidermal growth factor receptor 2 (HER2), and Ki-67; and axillary lymph node metastasis. According to ER, PR, and HER2 results, we classified the patients into hormone receptor (HR) positive or negative and HER2 positive or negative. Additionally for descriptive purposes, three DCIS subtypes were divided in supplementary analyses: hormone receptor (HR)-positive/HER2-negative (HR + HER2–), HER2-positive (HER2+), and HR-negative/Her2-negative (HR-HER2-) DCIS. All patients underwent mammography, ultrasound, and breast magnetic resonance imaging (MRI) before the curative surgery. Based on the imaging reports, clinical tumor size was defined as the largest tumor size among the mammography, ultrasound, and breast MRI assessments. Additionally, a suspicious axillary lymph node on radiologic evaluation was defined as the presence of an axillary lymph node with suspicious features in any one of the assessments (i.e., mammography, ultrasound, and breast MRI). We defined the following criteria as indicative of suspicious axillary lymph nodes: (i) cases where an axillary lymph node showed dense obliterated hila and cortical thickness visible on mammography, and (ii) cases where an axillary lymph node exhibited loss of the fatty hilum, a round shape, or eccentric cortical thickening on ultrasound or breast MRI. Among the patients with radiologically suspicious axillary lymph node, few patients (14 of 89 [15.7%]) underwent fine needle aspiration biopsy (FNAB) and all were confirmed negative.

### Statistical analyses

Our primary objective was to identify the axillary lymph node metastasis rate in patients with DCIS at diagnosis who underwent mastectomy with axillary surgery. We also assessed the upgrade rate of DCIS to invasive breast cancer. Axillary lymph node metastasis was defined as macrometastasis (size of most extensive metastatic lesion > 2 mm) and micrometastasis (size of most extensive metastatic lesion 0.2–2 mm) according to the American Joint Committee on Cancer guidelines (eighth edition). DCIS upgraded to invasive disease was defined as the diagnosis of invasive breast carcinoma in mastectomy specimens, including microinvasion.

Discrete variables according to axillary lymph node metastases and DCIS upgraded to invasive disease were compared using the chi-square or Fisher exact test. Univariable and multivariable analyses were performed using a binary logistic regression model to identify the predictive clinicopathologic features for axillary lymph node metastasis and DCIS upgrade to invasive disease. Odds ratio (OR) and 95% confidence interval (CI) with two-sided p-values were determined. Factors considered in the multivariable analysis included age (≤ 50 vs. > 50), Symptoms with a palpable mass or bloody nipple discharge (no vs. yes), clinical tumor size (as a continuous variable), radiologically suspicious axillary lymph node (no vs. yes), radiologically suspicious microcalcification (no vs. yes), nuclear grade (low vs. intermediate vs. high), HR status (negative vs. positive), HER2 status (negative vs. positive), and Ki-67 (< 14% vs. ≥ 14%). Data analyses were performed using SPSS version 25 (IBM Corp., Armonk, NY, USA), and a p-value of < 0.05 defined statistical significance.

## Results

### Baseline characteristics

Herein, we included 385 patients who were diagnosed with DCIS on preoperative biopsy and subsequently underwent a mastectomy. Table 1 summarizes the baseline clinicopathologic features. Overall, 165 patients (42.9%) were aged > 50 years, and the median clinical tumor size was 4.5 cm (range 0.5–12.9). Overall, 168 patients (43.6%) presented palpable mass or bloody nipple discharge before the diagnosis. In addition, 89 patients (23.1%) exhibited suspicious axillary lymph nodes on radiologic evaluation, and 266 (69.1%) displayed suspicious microcalcification on mammography. Within the available pathologic factors in biopsy specimens, high NG was identified in 86 of 323 (26.6%) patients, and comedo necrosis was detected in 194 (63.6%) of 305 patients. Considering postoperative factors, high NG was observed in 138 of 385 patients (35.8%), comedo necrosis in 290 of 381 (76.1%), and high Ki-67 expression in 106 of 380 (27.9%). Considering the 334 patients with available receptor status, 282 patients (73.2%) were HR positive and 127 (33.0%) were HER2 positive.

**Table 1:** Baseline characteristics

Variables		Total (*n* = 385)	
		N	(%)
Preoperative factors	Age (years)		
	≤ 50	220	57.1
	> 50	165	42.9
	Clinical tumor size, median (range), cm	4.5 (0.5–12.9)	
	≤ 2 cm	60	15.6
	2 –5 cm	172	44.7
	> 5 cm	153	39.7
	Palpable mass or bloody nipple discharge		
	No	217	56.4
	Yes	168	43.6
	Suspicious axillary lymph node on radiologic evaluation		
	No	296	76.9
	Yes	89	23.1
	Suspicious microcalcification on radiologic evaluation		
	No	119	30.9
	Yes	266	69.1
	Nuclear grade^*,†^		
	Low	53	16.4
	Intermediate	184	57
	High	86	26.6
	Comedo necrosis^*,†^		
	No	111	36.4
	Yes	194	63.6
Postoperative factors	Pathologic DCIS size, median (range), cm	4.15 (0.1–20)	
	Nuclear grade^‡^		
	Low	28	7.3
	Intermediate	219	56.9
	High	138	35.8
	Comedo necrosis^*,‡^		
	No	91	23.9
	Yes	290	76.1
	Hormone receptor ^*,‡^		
	Negative	99	25.7
	Positive	282	73.2
	HER2^*,‡^		
	Negative	254	66.0
	Positive	127	33.0
	Ki-67 (%)^*, ‡^		
	< 14	274	72.1
	≥ 14	106	27.9

^*^Missing values

^†^Values assessed in biopsy specimens

^‡^Values assessed in surgical specimens

DCIS = ductal carcinoma in situ, HR = hormone receptor, HER2 = human epidermal growth factor receptor 2

### Upgrade to invasive breast cancer

**Fig. 2:**
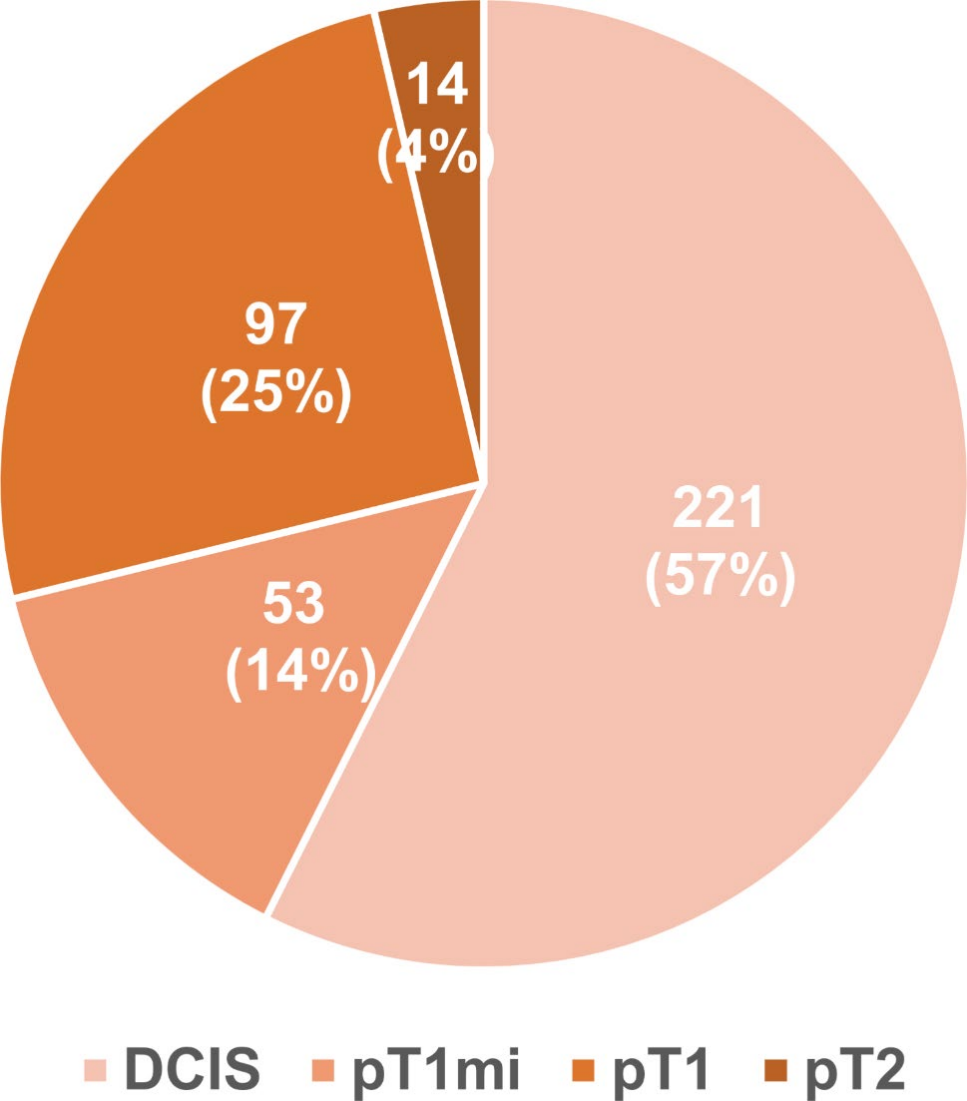
Pathologic results regarding the upgrade to invasive breast cancer. DCIS, ductal carcinoma in situ

Of the 385 patients, upgrade to invasive breast cancer was identified in 164 (42.6%): 53 (13.8%) were microinvasion, 97 (24.7%) were pT1, and 14 (3.6%) were pT2 stage (Fig. 2). Patients with DCIS upgraded to invasive cancer exhibited a larger clinical tumor size than those with pure DCIS (5.05 cm vs. 4.0 cm, *p* < 0.001; Table 2). Furthermore, patients with DCIS upgraded to invasive cancer showed a significantly higher proportion of radiologically suspicious axillary lymph nodes (31.7% vs. 16.7%, *p* = 0.001), NG 3 at biopsy (33.6% vs. 21.3%, *p* = 0.04), and palpable mass or bloody nipple discharge at presentation (57.3% vs. 33.5%, *p* < 0.001) than those with pure DCIS. On surgical specimen, lower rate of HR positive (65.9% vs. 78.7%, *p* = 0.003) and higher rate of high Ki-67 (37.8% vs. 19.9%, *p* < 0.001) were observed in patients with DCIS upgraded to invasive cancer. Regarding factors evaluated in surgical specimens, upgraded patients had a larger DCIS size and frequent comedo necrosis, along with high NG and Ki-67 expression (Supplementary Table 1).

**Table 2:** Baseline characteristics according to DCIS upgraded to invasive disease

Variables	DCIS (*n* = 221)		Upgrade to invasive disease (*n* = 164)		
	N	(%)	N	(%)	p-value
Age (y)					0.234
≤ 50	132	59.7	88	53.7	
> 50	89	40.3	76	46.3	
Palpable mass or bloody nipple discharge					< 0.001
No	147	66.5	70	42.7	
Yes	74	33.5	94	57.3	
Clinical tumor size, median (range), cm	4 (0.5–10.4)		5.05 (1.0–12.9)		< 0.001
Suspicious axillary lymph node on radiologic evaluation					0.001
No	184	83.3	112	68.3	
Yes	37	16.7	52	31.7	
Suspicious microcalcification on radiologic evaluation					0.295
No	73	33	46	28	
Yes	148	67	118	72	
Nuclear grade^*,†^					0.040
Low	34	18.6	19	13.6	
Intermediate	110	60.1	74	52.9	
High	39	21.3	47	33.6	
Comedo necrosis^*,†^					0.152
No	70	39.8	41	31.8	
Yes	106	60.2	88	68.2	
Hormone receptor^§^					0.003
Negative	44	19.9	55	33.5	
Positive	174	78.7	108	65.9	
HER2^§^					0.092
Negative	153	69.2	101	61.6	
Positive	65	29.4	62	37.8	
Ki-67 (%) ^§^					< 0.001
< 14%	174	78.7	100	61.0	
≥ 14%	44	19.9	62	37.8	

^*^Missing values

^†^Values assessed in biopsy specimens

^§^Values assessed in surgical specimens

Multivariable analysis (Table 3) revealed that the clinical tumor size (adjusted OR, 1.20; 95% CI, 1.06–1.35; *p* = 0.003), suspicious axillary lymph nodes on radiologic evaluation (adjusted OR, 2.01, 95% CI, 1.08–3.74; *p* = 0.028), and symptoms with a palpable mass or bloody nipple discharge (adjusted OR, 2.76; 95% CI, 1.64–4.64; *p* < 0.001) were independent factors for DCIS upgrade to invasive breast cancer. In patients without radiologically axillary lymph node metastasis, symptoms with a palpable mass or bloody nipple discharge (adjusted OR, 2.52, 95% CI, 1.40–4.54; *p* = 0.002), and high Ki-67 (adjusted OR, 2.14, 95% CI, 1.04–4.41; *p* = 0.040) were independent factors predictive of DCIS upgrade to invasive cancer (Supplementary Table 3).

**Table 3:** Odds ratio (OR) and 95% confidence interval (CI) for DCIS upgraded to invasive disease

Variables	Univariable		Multivariable	
	OR (95% CI)	p-value	OR (95% CI)	p-value
Age (years)				
> 50	Ref.		Ref.	
≤ 50	0.78 (0.52–1.17)	0.234	1.01 (0.58–1.76)	0.964
Palpable mass or bloody nipple discharge				
No	Ref.		Ref.	
Yes	2.67 (1.76–4.05)	< 0.001	2.76 (1.64–4.64)	< 0.001
Clinical tumor size, median (range), cm	1.26 (1.14–1.39)	< 0.001	1.20 (1.06–1.35)	0.003
Suspicious axillary lymph node on radiologic evaluation				
No	Ref.		Ref.	
Yes	2.31 (1.43–3.74)	0.001	2.01 (1.08–3.74)	0.028
Suspicious microcalcification on radiologic evaluation				
No	Ref.		Ref.	
Yes	1.27 (0.81–1.97)	0.296	1.43 (0.79–2.58)	0.232
Nuclear grade^*,†^		0.042		0.270
Low	Ref.		Ref.	
Intermediate	1.20 (0.64–2.27)	0.566	0.72 (0.31–1.68)	0.454
High	2.16 (1.07–4.36)	0.032	1.21 (0.42–3.46)	0.720
Comedo necrosis^*,†^				
No	Ref.		Ref.	
Yes	1.42 (0.88–2.29)	0.153	0.86 (0.45–1.66)	0.662
Hormone receptor^§^				
Negative	Ref.		Ref.	
Positive	0.50 (0.31–0.79)	0.003	0.69 (0.31–1.54)	0.368
HER2^§^				
Negative	Ref.		Ref.	
Positive	1.44 (0.94–2.22)	0.093	0.69 (0.32–1.46)	0.326
Ki-67 (%) ^§^				
< 14%	Ref.		Ref.	
≥ 14%	2.45 (1.55–3.88)	< 0.001	1.55 (0.82–2.93)	0.182

^*^Missing values

^†^Values assessed in biopsy specimens

^§^Values assessed in surgical specimens

### Axillary lymph node metastases

**Fig. 3:**
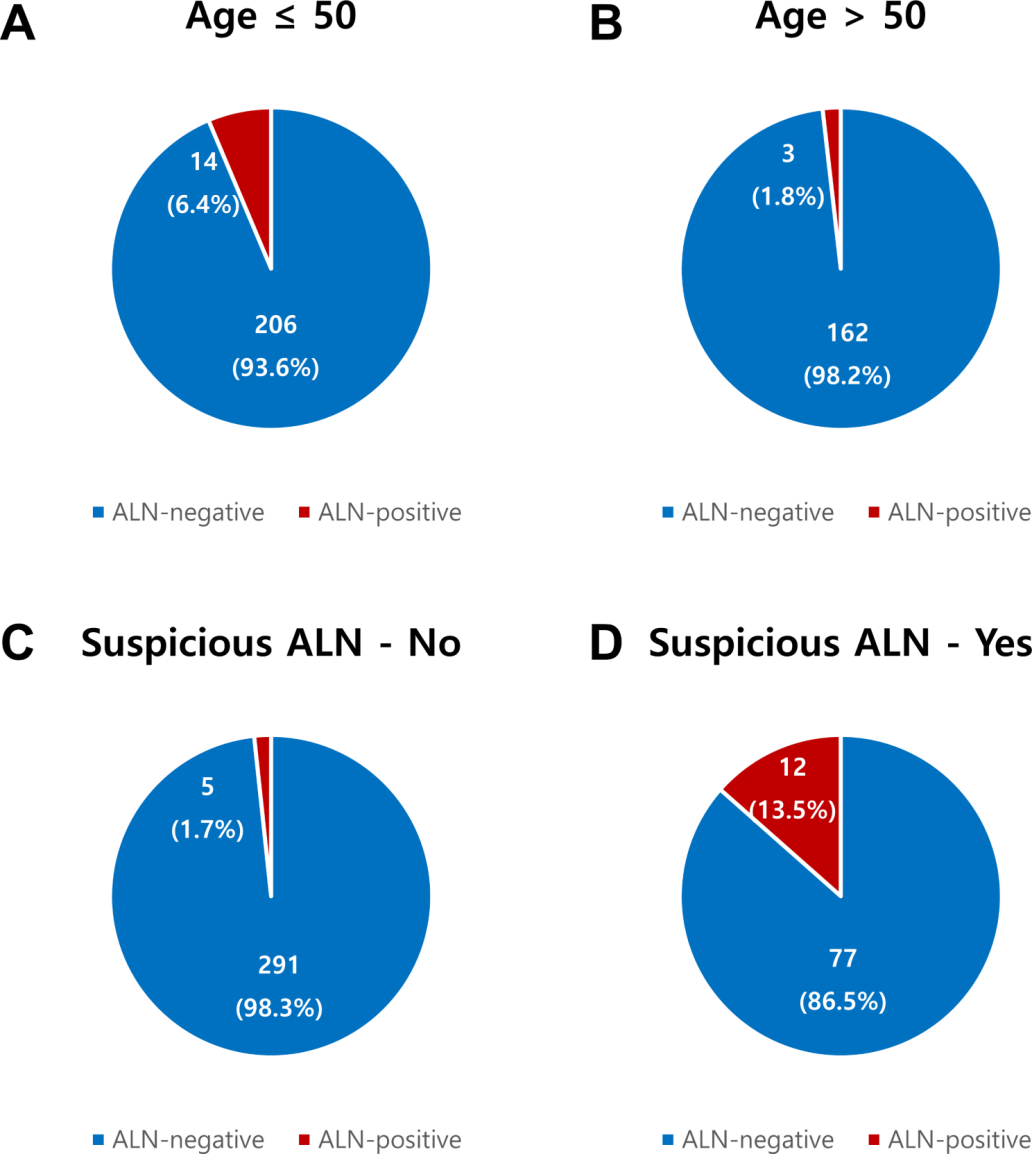
The rate of axillary lymph node (ALN) metastasis in (**A**) patients aged ≤ 50 (**B**) patients aged > 50, (**C**) patients without suspicious ALN on radiologic evaluation and (**D**) patients with suspicious ALN on radiologic evaluation. ALN, axillary lymph node

Overall, 17 of 385 (4.4%) patients had axillary lymph node metastases, all of which were pN1 stage. A higher proportion of patients with axillary lymph node metastases were aged ≤ 50 years (82.4% vs. 56%, *p* = 0.032; Table 4) and had suspicious axillary lymph nodes on radiologic evaluation (70.6% vs. 20.9%, *p* < 0.001). Considering postoperative characteristics, patients with axillary lymph node metastases exhibited a greater DCIS size than those without axillary lymph node metastases (6.5 cm vs. 4.0 cm, *p* = 0.002), with less frequent HER2 + subtype (12.5% vs. 40.6%, *p* = 0.011) (Supplementary Table 2). In the multivariable analysis, age ≤ 50 years (adjusted OR, 12.73, 95% CI, 1.18–137.51; *p* = 0.036; Table 5) and suspicious axillary lymph nodes on radiologic evaluation (adjusted OR, 9.31, 95% CI, 2.06–41.99; *p* = 0.004) were independent predictors for axillary lymph node metastases. Notably, the rate of axillary lymph node metastases was only 1.8% (3 of 165 patients) in patients aged > 50 years and 1.7% (5 of 196) in patients without suspicious axillary lymph nodes on radiologic evaluation (Fig. 3). When stratifying axillary lymph node metastasis by the two independent factors of age and suspicious axillary lymph node on radiologic evaluation (Table 6), only patients under the age of 50 with radiologically suspicious axillary lymph nodes had a high rate of pathologic axillary lymph node metastasis (24.4%). Patients over the age of 50 and/or without radiologically suspicious axillary lymph nodes had a low rate of lymph node metastasis (1.7–2.3%). Meanwhile, in patients without radiologically suspicious axillary lymph node on radiologic evaluation, univariable analysis showed that no clinicopathologic factors were associated with axillary lymph node metastasis (Supplementary Table 4).

**Table 4:** Baseline characteristics according to axillary lymph node metastasis

Variables	Node-negative (*n* = 368)		Node-positive (*n* = 17)		
	N	(%)	N	(%)	p-value
Age (y)					0.032
≤ 50	206	56	14	82.4	
> 50	162	44	3	17.6	
Palpable mass or bloody nipple discharge					0.197
No	210	57.1	7	41.2	
Yes	158	42.9	10	58.8	
Clinical tumor size, median (range), cm	4.5 (0.5–12.9)		5.1 (1.5–9.6)		0.112
Suspicious axillary lymph node on radiologic evaluation					< 0.001
No	291	79.1	5	29.4	
Yes	77	20.9	12	70.6	
Suspicious microcalcification on radiologic evaluation					> 0.999
No	114	31	5	29.4	
Yes	254	69	12	70.6	
Nuclear grade^*,†^					0.489^‡^
Low	52	16.8	1	7.7	
Intermediate	177	57.1	7	53.8	
High	81	26.1	5	38.5	
Comedo necrosis^*,†^					> 0.999^‡^
No	107	36.4	4	36.4	
Yes	187	63.6	7	63.6	
Hormone receptor^§^					0.500
Negative	96	26.1	3	17.6	
Positive	269	73.1	13	76.5	
HER2^§^					0.071
Negative	240	65.2	14	82.4	
Positive	125	34.0	2	11.8	
Ki-67 (%) ^§^					0.381
< 14%	264	71.7	10	58.8	
≥ 14%	100	27.2	6	35.3	

^*^Missing values

^†^Values assessed in biopsy specimens

^‡^The p-value was determined using Fisher’s exact test

^§^Values assessed in surgical specimens

**Table 5:** Odds ratio (OR) and 95% confidence interval (CI) for axillary lymph node metastasis

	Univariable		Multivariable	
	OR (95% CI)	p-value	OR (95% CI)	p-value
Age (y)				
> 50	Ref.		Ref.	
≤ 50	3.67 (1.04–12.99)	0.044	12.73 (1.18–137.51)	0.036
Palpable mass or bloody nipple discharge				
No	Ref.		Ref.	
Yes	1.90 (0.71–5.10)	0.203	0.95 (0.22–4.10)	0.940
Clinical tumor size, median (range), cm	1.18 (0.96–1.45)	0.114	1.44 (0.99–2.08)	
Suspicious axillary lymph node on radiologic evaluation				
No	Ref.		Ref.	
Yes	9.07 (3.10–26.52)	< 0.001	9.31 (2.06–41.99)	0.004
Suspicious microcalcification on radiologic evaluation				
No	Ref.		Ref.	
Yes	1.08 (0.37–3.13)	0.891	4.14 (0.45–37.83)	0.208
Nuclear grade^*,†^		0.525		0.290
Low	Ref.		Ref.	
Intermediate	2.06 (0.25–17.10)	0.505	2.93 (0.20–42.43)	0.431
High	3.21 (0.37–28.26)	0.293	11.14 (0.40–308.25)	0.155
Comedo necrosis^*,†^				
No	Ref.		Ref.	
Yes	1.00 (0.29–3.50)	0.998	0.41 (0.06–2.71)	0.357
Hormone receptor^§^				
Negative	Ref.		Ref.	
Positive	1.55 (0.43–5.54)	0.503	0.42 (0.03–5.19)	0.498
HER2^§^				
Negative	Ref.		Ref.	
Positive	0.27 (0.06–1.23)	0.090	0.30 (0.04–2.59)	0.276
Ki-67 (%) ^§^				
< 14%	Ref.		Ref.	
≥ 14%	1.58 (0.56–4.47)	0.385	0.62 (0.07–5.84)	0.676

^*^Missing values

^†^Values assessed in biopsy specimens

^§^Values assessed in surgical specimens

**Table 6:** Pathologic axillary lymph node metastasis by age and the presence of radiologically suspicious axillary lymph node

		Age	
		≤ 50	> 50
Suspicious axillary lymph node			
	Negative	3/175 (1.7%)	2/121 (1.7%)
	Positive	11/45 (24.4%)	1/44 (2.3%)

## Discussion

Surgical de-escalation is an important topic that is actively being discussed in the field of breast cancer. Although SLNB is a minimally invasive procedure, it is frequently associated with considerable short-term treatment-related upper limb morbidity, including lymphedema, pain, reduced range of motion, and muscle weakness [10, 11]. A growing body of literature indicates that SLNB can be omitted in patients initially diagnosed with DCIS [12–18]. Accordingly, the current guidelines suggest that axillary staging can be omitted in patients with DCIS undergoing breast-conserving surgery. Furthermore, several ongoing clinical trials are attempting to compare active surveillance to standard therapy in low-risk DCIS [19, 20]. Although DCIS has a less aggressive phenotype, most patients with DCIS who need mastectomy undergo SLNB owing to concerns regarding upgrade to invasive breast cancer and technical difficulty in performing SLNB after the removal of breast tissues. Thus, there is still an unmet need for axillary surgery omission in this subpopulation.

In this study, we investigated the incidence of upgraded pathologic stage from DCIS to invasive breast cancer and axillary lymph node metastasis in patients with DCIS who underwent mastectomy. Patients with DCIS upgrade to invasive cancer comprised approximately 42% of our study cohort, which was relatively higher than that in previous studies (21.8–37.1%) [15, 21, 22]. This discrepancy could be attributed to the inclusion of patients who underwent excision or breast-conserving surgery in previous studies, whereas ours predominantly focused on the patient population who underwent mastectomy. Consistent with the finding of a previous study [18], large tumor size and a palpable mass or bloody nipple discharge were risk factors for upgrade to invasive breast cancer.

Although the upgrade to invasive breast cancer occurred in a substantial number of patients, the actual axillary lymph node metastasis rate was only 4.4%. Consistently, a Danish group has speculated that the overall metastatic lymph node rate was < 9% in a nationwide study [17]. Another study revealed that 2.7% of patients who undergo mastectomy for DCIS had axillary lymph node metastasis on final pathology [16]. Moreover, similar to previous literature [18], we found that a younger age (≤ 50 years) and suspicious axillary lymph nodes on preoperative radiologic evaluation were independent predictors for axillary lymph node metastasis. The axillary lymph node metastasis rate was further reduced to 1.7–2.3% among females aged > 50 years and/or those lacking any suspicious axillary lymph nodes on radiologic evaluation. The recently published SOUND trial [23], although consisting of a different cohort of patients who underwent breast-conserving surgery for invasive breast cancer, showed that omitting SLNB in patients with early breast cancer did not result in inferior survival compared to the SLNB arm, with a primary 5-year distant disease-free survival rate of 98% vs. 97.7% (non-inferiority *p* = 0.02). In addition, the node-positive rate was relatively low (13.7%) in the SLNB arm. Nearly 95% of patients in the SOUND trial were classified as pT1. Similarly, 91% of patients who experienced an upgrade from DCIS to invasive breast cancer were also categorized as pT1 in our study. Considering the clinical implications of these findings, it may be safe to omit SLNB in patients with DCIS undergoing mastectomy if the aforementioned criteria are met. Ongoing studies on de-escalation of axillary surgery will provide further insights [24].

A novel surgical strategy may be an alternative approach in patients diagnosed with DCIS presenting the risk factors for axillary lymph node metastasis. Superparamagnetic iron oxide (SPIO) nanoparticles, an SLN tracer, showed comparable performance to the conventional radioisotope (Technetium 99m [Tc^99^]) and blue dye [25, 26]. The half-life of Tc^99^ is short (approximately 6 h), whereas SPIO nanoparticles can reside within the sentinel lymph nodes for a prolonged duration. This unique characteristic of SPIO nanoparticles could facilitate delayed SLNB as a secondary operation after primary breast surgery [27]. In the SentiNot trial, applying SPIO nanoparticles reduced unnecessary upfront SLNB by 78.3% in patients with a preoperative diagnosis of DCIS [28]. Among patients who underwent delayed SLNB, the SPIO nanoparticle group had higher sentinel lymph node detection rates than the Tc^99^ group. Considering only those patients who underwent mastectomy, the detection rates were 83.3 and 22.0% for the SPIO and Tc^99^ groups, respectively. Despite the requirement for a secondary surgical procedure and the limited adoption of SPIO, the option of a delayed SLNB with SPIO nanoparticle injection can be contemplated for patients with DCIS undergoing mastectomy, specifically those with age under 50 years and radiologically suspicious axillary lymph node.

Our study had several limitations. First, our study exclusively included patients who underwent mastectomy for DCIS in this study, potentially introducing selection bias. Moreover, due to the retrospective nature of the study, we could not identify cases where mastectomy was performed at the patient’s request. However, it’s worth noting that only about 15% of the patients (60 out of 385) were eligible for partial mastectomy for a tumor size smaller than 2 cm. Moreover, considering the median clinical tumor size was 4.5 cm, it is reasonable to assume that most patients likely underwent mastectomy due to factors such as large tumor size, multiple or widespread microcalcifications, or clinical nipple involvement. Consequently, clinical symptoms like palpable mass and bloody nipple discharge seemed to occur relatively frequently. Second, it is necessary to evaluate the usefulness of FNAB in predicting axillary lymph node metastasis. Given that FNAB-positive patients were classified as having invasive breast cancer, these cases were excluded. Moreover, the implementation rate of FNAB was low: patients without suspicious axillary lymph nodes on radiologic evaluation did not undergo FNAB, and only 14 of 89 (15.7%) patients with suspicious axillary lymph nodes on radiologic evaluation underwent FNAB. Of the 14 patients who underwent FNAB, one (7.1%) had axillary lymph node metastasis. Lastly, we cannot evaluate the oncologic safety of SLNB omission in these patients, as all patients received SLNB or SLNB followed by ALND in the current study. Hence, further prospective clinical trials are warranted to confirm the safety of omitting SLNB in the examined patient population.

In conclusion, although the axillary lymph node metastasis rate was low (4.4%), approximately 40% of patients with a preoperative diagnosis of DCIS who underwent mastectomy experienced upgrade to invasive breast cancer. Notably, axillary lymph node metastasis was rarely observed in patients aged > 50 years and/or in those without any suspicious lymph nodes on preoperative radiologic evaluation. Our real-world data suggest that the omission of SLNB may be feasible in these specific subpopulations. Further investigations with a prospective design and a more substantial sample size should be considered to comprehensively validate these findings.

### Electronic supplementary material

Below is the link to the electronic supplementary material.

Supplementary Material 1

## Data Availability

J. Jeong, corresponding author, had full access to all the data in the study and takes responsibility for the integrity of the data and the accuracy of the data analysis.
