# Supplementary material for: Selective omission of sentinel lymph node biopsy in mastectomy for ductal carcinoma in situ: identifying eligible candidates
Source: Breast Cancer Res. 2024 Apr 12;26:65. doi: 10.1186/s13058-024-01816-7 (PMC11015583; doi:10.1186/s13058-024-01816-7)
Supplement: Supplementary file 1 — Supplementary Material 1 [file 13058_2024_1816_MOESM1_ESM.docx]

| **Supplementary Table 1. Baseline characteristics evaluated in surgical specimens according to upgraded ductal carcinoma in situ** | | | | | |
| --- | --- | --- | --- | --- | --- |
|  | DCIS (n = 221) | | Invasive breast cancer (n = 164) | |  |
|  | N | (%) | N | (%) | p-value |
| Pathologic DCIS size, median (range), cm | 3.5 (0.1–20) | | 5.25 (0.6–20) | | <0.001 |
| Nuclear grade^*^ |  |  |  |  | <0.001 |
| Low | 25 | 11.3 | 3 | 1.8 |  |
| Intermediate | 128 | 57.9 | 91 | 55.5 |  |
| High | 68 | 30.8 | 70 | 42.7 |  |
| Comedo necrosis^*,†^ |  |  |  |  | <0.001 |
| No | 69 | 31.8 | 22 | 13.4 |  |
| Yes | 148 | 68.2 | 142 | 86.6 |  |
| Subtype^*,†^ |  |  |  |  | 0.056 |
| HR+HER2– | 110 | 61.5 | 83 | 53.5 |  |
| HER2+ | 67 | 37.4 | 64 | 41.3 |  |
| HR-HER2- | 2 | 1.1 | 8 | 5.2 |  |
| Ki-67 (%)^*,†^ |  |  |  |  | <0.001 |
| <14 | 174 | 79.8 | 100 | 61.7 |  |
| ≥14 | 44 | 20.2 | 62 | 38.3 |  |

^*^Values assessed in surgical specimens.

^†^Missing values

DCIS = ductal carcinoma in situ, HR = hormone receptor, HER2 = human epidermal growth factor receptor 2

| **Supplementary Table 2. Baseline characteristics evaluated in surgical specimens according to axillary lymph node metastasis** | | | | | |
| --- | --- | --- | --- | --- | --- |
|  | Node-negative (n = 368) | | Node-positive (n = 17) | |  |
|  | N | (%) | N | (%) | p-value |
| Pathologic DCIS size, median (range), cm | 4 (0.1–20.0) | | 6.5 (1.7–12.0) | | 0.002 |
| Nuclear grade^*^ |  |  |  |  | 0.476 |
| Low | 28 | 7.6 | 0 | 0 |  |
| Intermediate | 208 | 56.5 | 11 | 64.7 |  |
| High | 132 | 35.9 | 6 | 35.3 |  |
| Comedo necrosis^*,†^ |  |  |  |  | 0.381^‡^ |
| No | 89 | 24.5 | 2 | 11.8 |  |
| Yes | 275 | 75.5 | 15 | 88.2 |  |
| Subtype^*,†^ |  |  |  |  | 0.011 |
| HR+HER2– | 181 | 56.9 | 12 | 75 |  |
| HER2+ | 129 | 40.6 | 2 | 12.5 |  |
| HR-HER2- | 8 | 2.5 | 2 | 12.5 |  |
| Ki-67 (%)^*,†^ |  |  |  |  | 0.398^‡^ |
| <14 | 264 | 72.5 | 10 | 62.5 |  |
| ≥14 | 100 | 27.5 | 6 | 37.5 |  |

^*^Values assessed in surgical specimens.

^†^Missing values

^‡^The p-value was obtained using Fisher’s exact test.

DCIS = ductal carcinoma in situ, HR = hormone receptor, HER2 = human epidermal growth factor receptor 2

| **Supplementary Table 3. Odds ratio (OR) and 95% confidence interval (CI) for DCIS upgraded to invasive disease in patients without suspicious axillary lymph node** | | | | |
| --- | --- | --- | --- | --- |
|  | Univariable | | Multivariable | |
| Variables | OR (95% CI) | p-value | OR (95% CI) | p-value |
| Age (years) |  |  |  |  |
| >50 | Ref. |  | Ref. |  |
| ≤50 | 0.78 (0.48–1.25) | 0.304 | 0.88 (0.48–1.64) | 0.695 |
| Palpable mass or bloody nipple discharge |  |  |  |  |
| No | Ref. |  | Ref. |  |
| Yes | 2.51 (1.54–4.07) | <0.001 | 2.52 (1.40–4.54) | 0.002 |
| Clinical tumor size,  median (range), cm | 1.17 (1.05–1.31) | 0.004 | 1.11 (0.97–1.27) | 0.143 |
| Suspicious microcalcification on radiologic evaluation |  |  |  |  |
| No | Ref. |  | Ref. |  |
| Yes | 1.27 (0.76–2.12) | 0.359 | 1.24 (0.64–2.43) | 0.528 |
| Nuclear grade^*,†^ |  | 0.132 |  | 0.154 |
| Low | Ref. |  | Ref. |  |
| Intermediate | 0.87 (0.43–1.76) | 0.699 | 0.49 (0.19–1.25) | 0.134 |
| High | 1.63 (0.73–3.64) | 0.232 | 0.83 (0.25–2.77) | 0.759 |
| Comedo necrosis^*,†^ |  |  |  |  |
| No | Ref. |  | Ref. |  |
| Yes | 1.44 (0.83–2.51) | 0.194 | 1.11 (0.52–2.35) | 0.789 |
| Hormone receptor^§^ |  |  |  |  |
| Negative | Ref. |  | Ref. |  |
| Positive | 0.56 (0.32–0.97) | 0.040 | 0.89 (0.33–2.35) | 0.808 |
| HER2^§^ |  |  |  |  |
| Negative | Ref. |  | Ref. |  |
| Positive | 1.52 (0.91–2.54) | 0.107 | 0.84 (0.34–2.09) | 0.706 |
| Ki-67 (%)^§^ |  |  |  |  |
| < 14% | Ref. |  | Ref. |  |
| ≥ 14% | 2.42 (1.39–4.20) | 0.002 | 2.14 (1.04–4.41) | 0.040 |

^*^Missing values

^†^Values assessed in biopsy specimens.

^§^Values assessed in surgical specimens.

**Supplementary Table 4. Odds ratio (OR) and 95% confidence interval (CI) axillary lymph node metastasis in patients without suspicious axillary lymph node**

|  | Univariable | |
| --- | --- | --- |
|  | OR (95% CI) | p-value |
| Age (y) |  |  |
| >50 | Ref. |  |
| ≤50 | 1.04 (0.17–6.31) | 0.968 |
| Palpable mass or bloody nipple discharge |  |  |
| No | Ref. |  |
| Yes | 2.30 (0.38–13.95) | 0.367 |
| Clinical tumor size, median (range), cm | 1.13 (0.78–1.65) | 0.525 |
| Suspicious microcalcification on radiologic evaluation |  |  |
| No | Ref. |  |
| Yes | 0.30 (0.50–1.85) | 0.196 |
| Nuclear grade^*,†^ |  |  |
| Low | Ref. |  |
| Intermediate | N/E | 0.998 |
| High | N/E | 0.998 |
| Comedo necrosis^*,†^ |  |  |
| No | Ref. |  |
| Yes | 1.27 (0.11–14.18) | 0.847 |
| Hormone receptor^§^ |  |  |
| Negative | Ref. |  |
| Positive | N/E | 0.997 |
| HER2^§^ |  |  |
| Negative | Ref. |  |
| Positive | 0.60 (0.07–5.42) | 0.647 |
| Ki-67 (%)^§^ |  |  |
| < 14% | Ref. |  |
| ≥ 14% | N/E | 0.997 |

^*^Missing values

^†^Values assessed in biopsy specimens.

^§^Values assessed in surgical specimens

N/E: Not evaluable
